# Antigen-Specific Suppression and Immunological Synapse Formation by Regulatory T Cells Require the Mst1 Kinase

**DOI:** 10.1371/journal.pone.0073874

**Published:** 2013-09-09

**Authors:** Takashi Tomiyama, Yoshihiro Ueda, Tomoya Katakai, Naoyuki Kondo, Kazuichi Okazaki, Tatsuo Kinashi

**Affiliations:** 1 Division of Gastroenterology and Hepatology, the Third Department of Internal Medicine, Kansai Medical University, Hirakata, Osaka, Japan; 2 Department of Molecular Genetics, Institute of Biomedical Science, and Core Research for Engineering, Science and Technology, Japan Science and Technology Agency, Kansai Medical University, Hirakata, Osaka, Japan; Centre de Recherche Public de la Santé (CRP-Santé), Luxembourg

## Abstract

Although the cell-to-cell contact between CD4^+^Foxp3^+^ regulatory T (Treg) and their target cells is important for the suppressor function of Treg cells, the regulation of this process is not well understood. Here we show that the Mst1 kinase plays a critical role in the suppressor function of Treg cells through regulation of cell contact dependent processes. *Mst1* ^*-/-*^ Treg cells failed to prevent the development of experimental colitis and antigen-specific suppression of naïve T cells proliferation *in vitro*. *Mst1* ^*-/-*^ Treg cells exhibited defective interactions with antigen-presenting dendritic cells (DCs), resulting in reduced down-regulation of costimulatory molecules. While wild-type CD4^+^ Foxp3^+^ Treg cells formed mobile immunological synapses on supported planar membrane, *Mst1* ^*-/-*^ Treg cells did not exhibit ICAM-1 ring or central peptide-MHC clustering. Using two-photon imaging we showed that antigen-specific wild-type Treg cells exhibited dynamic mobile contacts with antigen-pulsed DCs bearing stably associated naïve T cells. In contrast, *Mst1* ^*-/-*^ Treg had impairments in their interactions with DCs. Thus, Mst1 is required for Treg cells to mediate contact-dependent suppressor functions.

## Introduction

Regulatory T (Treg) cells exert suppressor function in T cell responses to self-antigen, microbial pathogens, transplants, and tumors. Treg cell–mediated suppression in the priming and effector phases of T cell responses involves cell-to-cell contact-dependent process as well as bystander suppression [1,2]. Treg cells act on antigen-presenting dendritic cells (DCs) by inhibiting their function through down-modulation of co-stimulatory molecules [3,4] or by inducing perforin-dependent cell death [5]. Intravital two-photon imaging has shown that the absence of Treg cells prolongs contact duration between DCs and T cells specific for self-antigens [6,7], tumor-related antigens [5], and foreign antigens [8]. Thus, Treg cells can inhibit antigen-induced stable contacts between T cells and DCs, thereby suppressing self-reactive T cells and low-avidity T-cell priming. Adoptively transferred *in vitro*–expanded islet–specific Treg cells were shown to stably interact with DCs in pancreatic lymph nodes (LN), similar to activated T_H_ cells [7]. However, it remains unclear how natural Treg cells interact with antigen-loaded DCs to suppress T cell priming.

An *in vitro* study showed that antigen-specific natural Treg cells formed conjugates with antigen-loaded DCs more efficiently than naïve T cells with the same specificity, suggesting that Treg cells could outcompete naïve T cells for antigen-loading DCs, thereby suppressing T cell priming [9]. The conjugate of Treg cells and DCs formed via LFA-1/ICAM-1-dependent adhesion was required for the suppressor function of Treg cells [9]. Indeed, LFA-1 is more highly expressed in Treg cells than naïve T cells and it is required for efficient generation and function of Treg cell in mice [10,11] and in humans [12]. The immunological synapse (IS) refers to molecular organization of the antigen-specific T cell–APC contact area and it is characterized with segregation of central TCR/pMHC (central supramolecular activation cluster, cSMAC) and peripheral LFA-1/ICAM-1 (pSMAC) [13], which can be reproduced on supported planar membrane [14,15]. Proper organization and stability of IS are regulated by several factors. PKCθ localized in cSMAC destabilizes the IS and promotes mobile synapse in naïve T cells [16], whereas PKCθ is sequestered away from the IS of Treg cells and inhibits Treg suppressor function [17]. Talin, a critical regulator of integrin-dependent adhesion [18], are associated with pSMAC. Talin1 deficiency severely diminishes antigen-specific T-dC interactions through abrogation of LFA-1 and ICAM-1 interaction, but LFA-1 cluster at T-dC contacts remains intact [19]. Although LFA-1 has been well characterized in multiple immune functions including lymphocyte homing, antigen-dependent T cell–antigen presenting cell (APC) interactions, and cytotoxicity, elucidation of the regulatory mechanisms of LFA-1 in Treg cells is still limited.

We previously showed that Mst1, the mammalian homolog of Drosophila Hpo, interacted with the Rap1 binding protein RAPL and transmitted signals that chemokine-induced LFA-1-dependent lymphocyte adhesion and TCR-dependent adhesion and immunological synapse formation with APC [20]. *Mst1* ^*-/-*^ mice showed defective lymphocyte trafficking and thymocyte egress [21–23]. In addition, *Mst1* ^*-/-*^ mice exhibited inefficient thymocyte selection and autoimmune-like disorders with age, suggesting that Mst1-regulation of LFA-1 play an important role in the maintenance of self-tolerance [24]. To elucidate the role of Mst1 in Treg cells, we investigated whether Mst1 deficiency affect the suppressor function and adhesive behaviors of Treg cells. We found that Mst1 is required for the suppression of T cell–induced colitis and T cell proliferation *in vitro* by natural Foxp3^+^ CD4^+^ Treg cells. Two-photon imaging within LN tissues and immunological synapse formation on supported planar membrane revealed the dynamic interactions of Treg cells with antigen-loaded DCs with mobile immunological synapse (IS) formation. *Mst1* ^*-/-*^ Treg cells are severely impaired in interactions with DCs, which is characterized by the defect of both cSMAC and pSMAC formations, therefore indicating that Mst1 plays a crucial role in shaping Treg cell suppressor functions by regulating their adhesive behaviors.

## Materials and Methods

### Mice

*Mst1* ^*-/-*^ mice on a C57BL/6 background were previously described [21]. C57BL/6 mice were purchased from CLEA Japan. OT-II mice on a C57BL/6 background [25] were obtained from Jackson Laboratories. Foxp3-IRES-GFP knock-in (Foxp3-GFP) mice [26] were kindly provided by Dr. M. Hori (RIKEN, Yokohama, Japan). Mst1^-/-^, OT-II, and Foxp3-GFP mice were crossed to generate Mst1^-/-^ OT-II mice expressing Foxp3-GFP. RAG2^-/-^ mice [27] were obtained from Center for Animal Resources and Development, Kumamoto University (Kumamoto, Japan). All mice were maintained under specific pathogen–free conditions in the animal facility at Kansai Medical University (Osaka, Japan) and were treated and used for experiments in accordance with institutional guidelines and ethical approvals for the care of experimental animals. The protocols were approved by the Animal Care and Use Committee of Kansai Medical University (#13-015).

### Antibodies and reagents

FITC-, PE-, PerCP-Cy5.5-, or APC-conjugated antibodies specific to CD4 (GK1.5, RM4-4), CD8 (53-6.7), CD11a (M17/4), CD11c (N418), CD18 (M18/2), CD44 (IM7), CD62L (MEL-14), FoxP3 (FJK-16s), Vα2(B20.1), and Vβ5(MR9-4) were purchased from eBioscience (San Diego, CA) or BD Bioscience (San Jose, CA). carboxyfluorescein diacetate, succinimidyl ester (CFSE), (5-(and-6)-(((4-Chloromethyl) Benzoyl) Amino) Tetramethylrhodamine (CMTMR), or 7-Amino-4-Chloromethylcoumarin (CMAC) for cell labeling were obtained from Invitrogen (Grand Island, NY). OVA_323-339_ peptides (ISQAVHAAHAEINEAGR) were obtained from Anaspec (Fremont, CA).

### Cell preparation

T cells were isolated from spleens and lymph nodes of 12- to 20-wk-old mice by magnetic-activated cell sorting using a pan-T cell isolation kit II (Miltenyi Biotec, Auburn, CA). Isolated T cells were stained with APC-labeled anti-CD8, PerCP-Cy5.5 labeled anti-CD62L antibodies. CD62L^hi^CD8^-^Foxp3-GFP^-^ or CD8^-^Foxp3-GFP^+^ cells were sorted as naïve T or Treg cells, respectively, by using a FACS Aria cell sorter (BD Bioscience). The purity of naïve T and Treg cells were greater than 97%. For preparation of CD11c^+^ dendritic cells, spleens of C57BL/6 mice were digested with collagenase D (1 mg/ml) and DNase I (50µg/ml) for 30 min at 37°C and CD11c^+^ cells were isolated by magnetic-activated cell sorting using anti-CD11c beads (Miltenyi Biotec). Dendritic cells were generated *in vitro* from bone marrow cells of C57BL/6 mice with optimal concentration of GM-CSF-conditioned medium as described [28].

### Colitis model

*Rag2* ^*-/-*^ mice were adoptively transferred with 5×10^5^ naïve T cells with or without 1×10^5^ Treg cells [29]. Mice were monitored weekly by body weight. At 10 weeks mice were sacrificed, and colon specimens were fixed in 10% formalin, paraffin-embedded, sectioned, and stained with hematoxylin and eosin. Digital images of organs were acquired by Nanozoomer (Hamamatsu Photonics, Hamamatsu, Japan).

### In vitro suppression assay

For the antigen-independent suppression assay, naïve T cells (2.5×10^4^) and Treg cells (0-2.5×10^4^) prepared from wild-type and *Mst1* ^*-/-*^ mice as described above, were co-cultured at different ratios in the presence of CD11c^+^ DCs (2.5×10^3^) supplemented with anti-CD3 (1 µg/ml) (145-2C11, BD bioscience) for 3 days in 96-well round-bottomed plates (BD, Franklin Lakes, NJ). [^3^H] thymidine (1 µCi per well; PerkinElmer) was added during the last 8 h of culture. For the antigen-specific suppression assay, naïve T cells and Treg cells prepared from *Mst1* ^*+/+*^OT-II and *Mst1* ^*-/-*^ OT-II mice were used and stimulated with 0.1 µM OVA_323-339_ peptide. In some cases, the cells were harvested for staining of CD86, and analyzed using a FACS caliber flow cytometer (BD bioscience).

### In vitro conjugation assays

CFSE- or CMTMR-labeled naive OT-II T cells (1×10^5^) and Treg cells (1×10^5^) prepared from wild-type and *Mst1* ^*-/-*^ OT-II mice as described above, were co-cultured in the presence of CD11c^+^ splenic DCs (1×10^4^) supplemented with OVA_323–339_ peptides for 16 h in multi-well glass bottom dishes (Matsunami, Osaka, Japan) coated with fibronectin. Images were acquired using a confocal microscope (LSM510-META, Zeiss).

### Immunological synapse assay

Mouse ICAM-1-GPI was purified, fluorescently labeled with Cy3, and incorporated into liposomes consisting of 0.4 mM DOPC containing 0.1 mol% biotin-CAP-PE (Avanti Polar Lipids, Alabaster, AL) essentially as described [30]. ICAM-1 liposomes were mixed with DOPC/CAP-biotin-PE liposomes containing 1 mol% DGS-NTA-Ni at a 2:1 ratio and deposited onto piranha-treated cover glass mounted on a 35 mm dish. Lipid bilayers on the glass were blocked with 1% BSA and loaded sequentially with Alexa488-labeled streptavidin (1 µg/ml) and mono-biotinylated I-A^b^ with 0.1 M OVA peptide (AAHAEINEA) obtained through the NIH Tetramer Core Facility (Atlanta, GA) and CD80-histidine tag (Creative Biomart, Shirley, NY). Densities of ICAM-1 (250 molecules/μm^2^) and CD80 (90 molecules/μm^2^) were measured as described [30], and supported pMHC-dependent attachment and IS formation on lipid bilayers. Treg cells (1×10^5^) in phenol-red free RPMI-1640/5%FCS were added to lipid bilayers, and live-cell imaging was performed in a humidified 5% CO_2_ incubator chamber on the stage of an inverted Olympus IX81 microscope (Olympus, Tokyo, Japan) equipped with a Hamamatsu C9100-13 EM-CCD camera and 60× objective lens (NA 1.45). Time-lapse images were acquired for peptide-MHC in the epi-illumination mode and for ICAM-1 in the TIRF mode with a 564 nm solid-state laser (Andor, Belfast, UK). All data were collected and analyzed using Metamorph software (Molecular Devices, Sunnyvale, CA).

### Two-photon imaging using LN slices

CD11c^+^ BMDCs (1×10^6^) were stimulated with 1 µg/ml lipopolysaccharide for 16 h and labeled with CFSE prior to loading with or without 1 µM OVA_323-339_, and were subcutaneously injected into the front paw of C57BL/6 mice. After 24 h, brachial LNs were isolated, embedded in 4% low-melting agarose gels, and then cut open with a vibratome (DSK, Kyoto, Japan). OT-II naïve T cells, *Mst1* ^*+/+*^ or *Mst1* ^*-/-*^ OT-II Treg cells were labeled with CMAC or CMTMR. The mixture of these cells was applied to the cut LN surface. After incubation for 30 min at 37°C/5% CO_2_, the LN slice was mounted on a RC-26 flow chamber (Warner Instruments, Hamden, CT), with a supply of warm RPMI1640 medium saturated with a 95% O_2_/5% CO_2_ gas. Two-photon imaging was set up using a FV1000 upright microscope (Olympus), with a Ti: sapphire laser (MaiTai HP DeepSee-OL, Spectra-Physics, Irvine, CA) and a 25X/1.05 NA objective (XLPLN25XWMP, Olympus). The images were taken with excitation wavelength at 820 nm. Emitted light was passed through 420–460 nm, 495–540 nm, and 575–630 nm bandpass filters to detect CMAC, CFSE, and CMTMR fluorescence, respectively. Time-lapse x-y images with 2.5 µm apart of z-stacks were taken every 20 sec for 20 min. Volocity software (PerkinElmer, Waltham, MA) was used for 3D cell tracking and calculation of cell motility parameters.

## Results

### Defective suppressor functions of Mst1^-/-^ Treg cells

**Figure 1:**
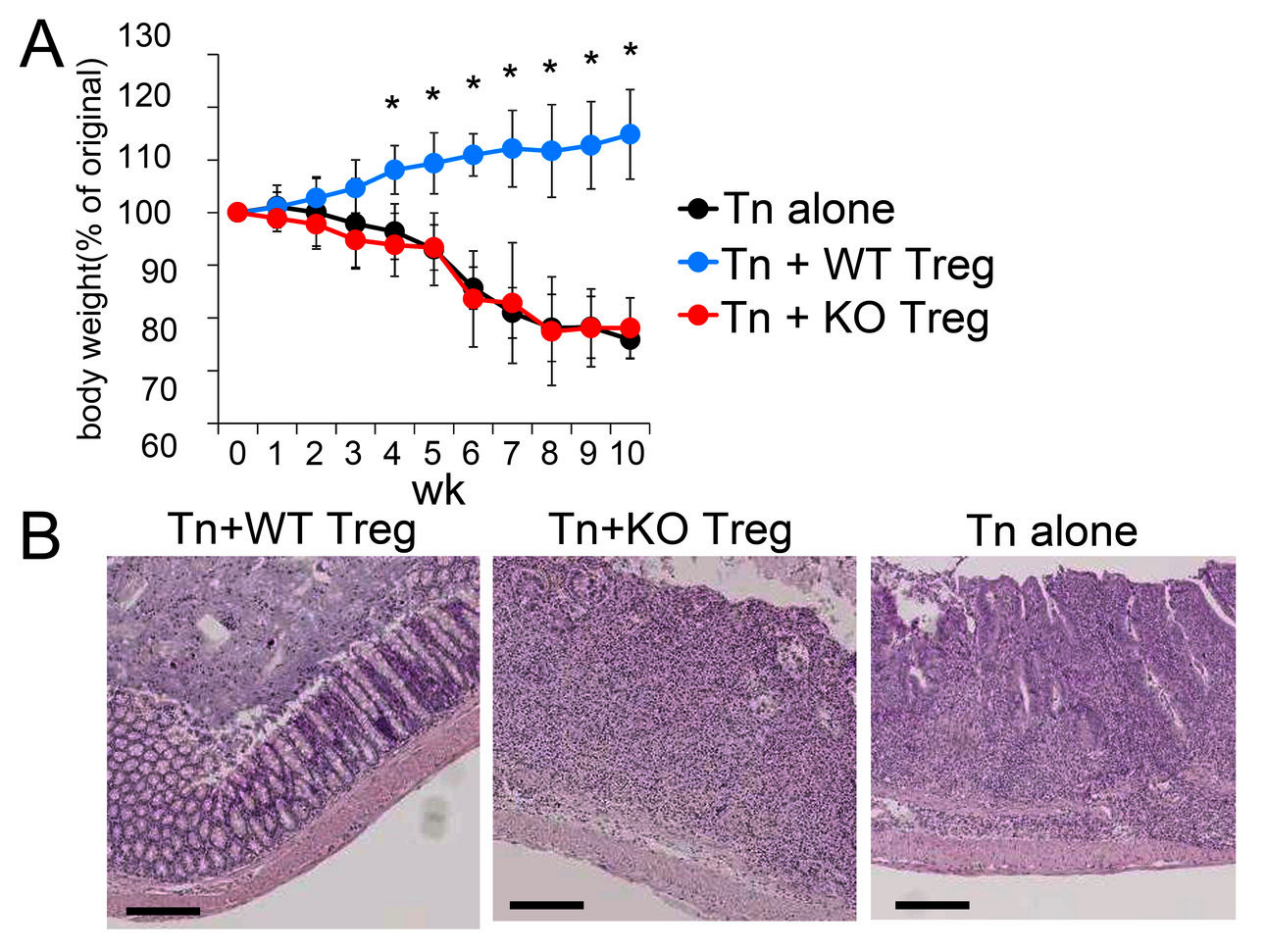
*Mst1* ^*-/-*^ Treg cells have impaired suppressor activity in a colitis murine model. CD4^+^ Foxp3^-^ CD62L^hi^ naïve T (Tn) cells were adoptively transferred alone or in combination with CD4^+^ Foxp3^+^ *Mst1* ^*+/+*^ (WT) or CD4^+^ Foxp3^+^ *Mst1* ^*-/-*^ (KO) Treg cells into *Rag2* ^*-/-*^ mice. (**A**) Changes in body weight after transfer. Data (n=4 per group) are from 4 independent experiments (mean ± SD). P-values were calculated by Mann–Whitney U-test, *p<0.05. (**B**) Representative colon histology in (A). Bars indicate 250 µm.

In *Mst1* ^*-/-*^ mice, Foxp3^+^ CD4^+^ Treg cells in the thymus were decreased but recovered in the periphery [24] and exhibited comparable levels of Foxp3 and surface receptors including CTLA-4 (Figure **S1**). To determine whether the Mst1 deficiency altered Foxp3^+^ Treg cell functions, we employed the experimental colitis model induced by adoptive transfer of naïve T cells into *Rag2* ^*-/-*^ mice. In this model, the co-transfer of Treg cells into lymphopenic mice prevents the colitis [31]. Indeed, defective weight gain (Figure **1A**) and development of the colitis (Figure **1B**) were induced by adoptive transfer of CD62L^hi^ naïve T cells, but these symptoms were attenuated by co-transfer of Foxp3^+^ Treg cells. In contrast, *Mst1* ^*-/-*^ Foxp3^+^ Treg cells were not able to prevent colitis and showed a similar degree of the body weight loss as mice that received only naïve T cells. Histological examination confirmed that the mice that received naïve T cells and *Mst1* ^*-/-*^ Treg cells exhibited intestinal inflammation with loss of crypts and infiltration of leukocytes, resulting in intestinal thickening and shortening (Figure **1A, 1B**).

**Figure 2:**
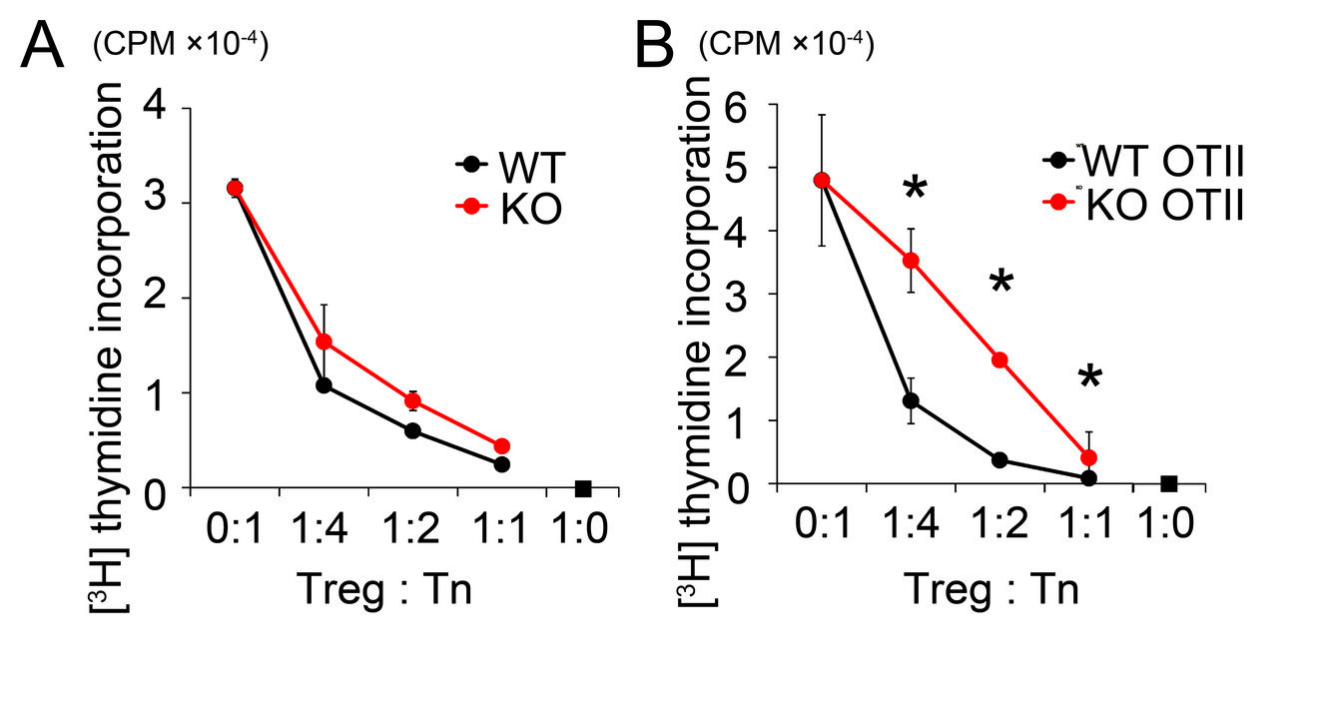
Antigen-specific suppression is impaired in *Mst1* ^*-/-*^ Treg cells *in vitro*. (**A**) T-cell suppression assays with Treg cells from *Mst1* ^*+/+*^ (WT) and *Mst1* ^*-/-*^ (KO) mice in the presence of 1 µg/ml of anti-CD3. The panel shows the results (n=3 per group) from 3 independent experiments. (**B**) T-cell suppression assays with Treg cells from OT-II WT and OT-II KO mice in the presence of 0.1 µM OVA_323–339_. The panel shows the results (n=3 per group) from 3 independent experiments. P-values were calculated by Mann–Whitney U-test, *p<0.05; **p<0.01.

Since Mst1 is required for lymphocyte trafficking, the diminished ability of *Mst1* ^*-/-*^ Treg cells to suppress experimental colitis may be due to the impaired trafficking of *Mst1* ^*-/-*^ Treg cells. To confirm defective suppressor functions of *Mst1* ^*-/-*^ Treg cells directly, we examined *in vitro* suppression of naïve T cell proliferation. Both wild-type and *Mst1* ^*-/-*^ Treg cells suppressed naïve T cell proliferation comparably in response to antigen-independent stimulation using anti-CD3 antibody (Figure **2A**). To evaluate antigen-specific suppression, we prepared Treg cells and naïve T cells from *Mst1* ^*+/+*^ OT-II or *Mst1* ^*-/-*^ OT-II mice. Compared to *Mst1* ^*+/+*^ OT-II Treg cells, *Mst1* ^*-/-*^ Treg cells were less efficient at mediating the suppression of naïve OT-II CD4^+^ T cell proliferation upon stimulation with OVA peptide and splenic DCs (Figure **2B**). Since Mst1-deficiency did not influence on anti-CD3-induced suppressor function, Mst1 is dispensable for the downstream events of TCR stimulation in Treg cells, instead it is more likely required for the contacts between Treg cells and antigen-loaded DCs.

### Impairments of antigen-dependent conjugate formation of Mst1^-/-^ Treg cells

**Figure 3:**
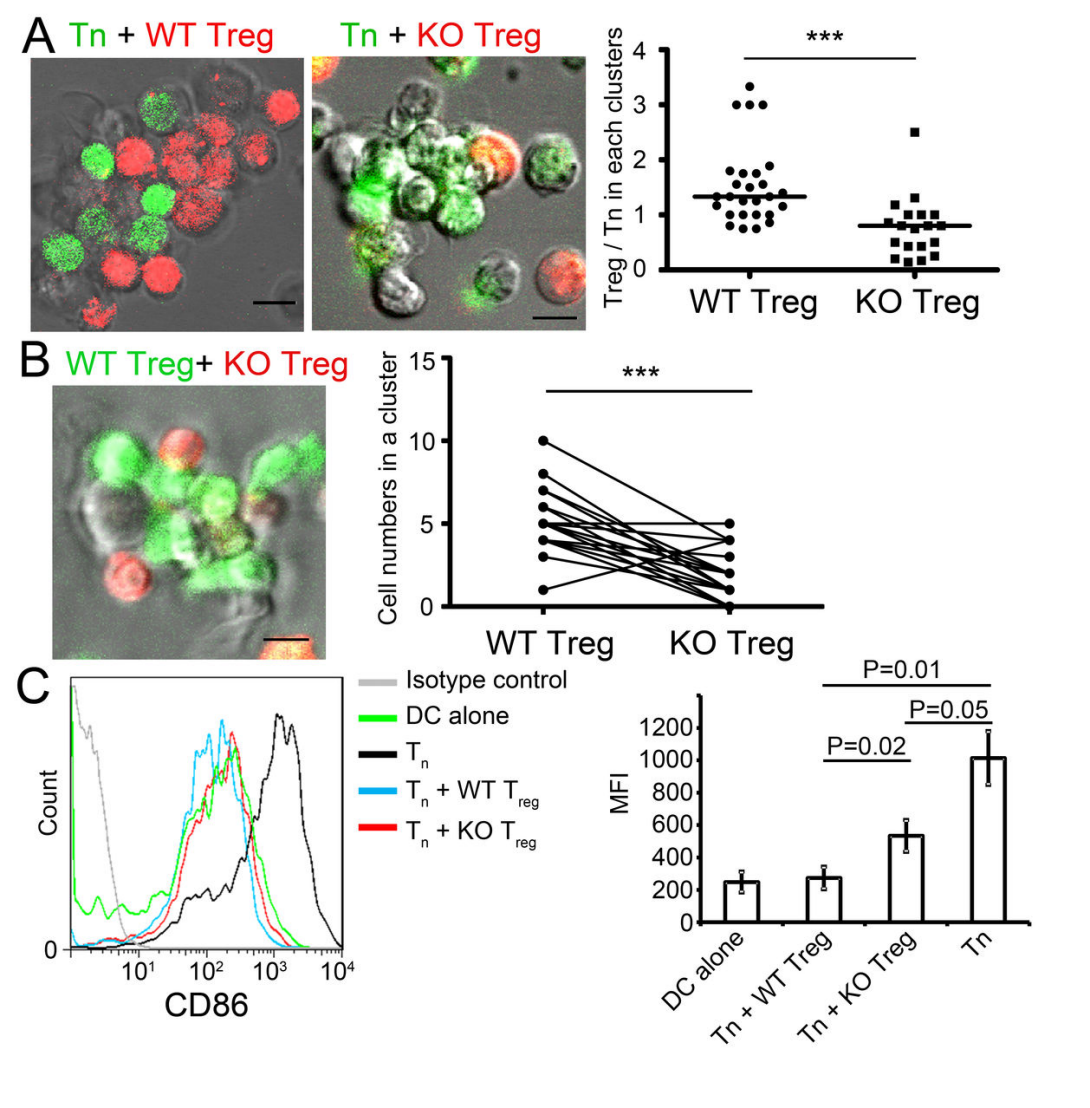
*Mst1* ^*-/-*^ Treg cells exhibited inefficient conjugate formation and defective down-regulation of CD86 with DCs. (**A**) CFSE-labeled naïve T cells (Tn, green) and CMTMR-labeled *Mst1* ^*+/+*^(WT, left) or *Mst1* ^*-/-*^ (KO, right) WT and KO Treg cells (red) obtained from OT-II mice were mixed at a 1:1 ratio and cultured for 16 h with CD11c^+^ splenic DCs in the presence of 1 µM OVA_323–339_. The right panel shows the ratio of Treg/Tn cell number in each cluster. Data (WT, n= 27; KO, n= 19) were derived from 3 independent experiments. P-values were calculated by Mann–Whitney U-test, ***p<0.0001. (**B**) OT-II WT Treg cells (green) and CMTMR-labeled OT-II KO Treg cells (red) were mixed at a 1:1 ratio and cultured for 16 h with C57BL/6 splenic DCs in the presence of 1 µM OVA_323–339_ (left). The right panel shows the cell number in each cluster. Data (n=22) were derived from 3 independent experiments. P-values were calculated by paired t-test, ***p<0.0001. (**C**) CD86 down-regulation was impaired in OT-II KO Treg cells. Tn and Treg cells were mixed at a 1:1 ratio with splenic DCs in the presence of 1 µM OVA_323–339_ and were cultured for 3 days. CD86 expression on DCs was measured using flow cytometry. The histogram (left) represents 3 independent experiments. The bar graph (right) represents the mean fluorescence intensity (n=3 per group) with SD from 3 independent experiments with P-values by Student t-test indicated.

To examine this possibility, we co-cultured OT-II Foxp3^+^ CD4^+^ Treg cells and OT-II CD62L^hi^ Foxp3^-^ CD4^+^ naïve T cells with CD11c^+^ splenic DCs. When mixed with an equal ratio of wild-type Treg and naive T cells, they formed conjugates with the OVA peptide–pulsed DCs (Figure **3A**). This antigen-specific conjugate formation was inhibited almost completely with antibodies to LFA-1 and ICAM-1 (not depicted), as reported [9]. Wild-type Treg cells formed conjugates with DCs more efficiently than naïve T cells; the relative number of Treg cells in each cluster ranged from 0.75 to 3.33 with a median of 1.33, which is in agreement with the previous study [9]. On the other hand, *Mst1* ^*-/-*^ OT-II Treg cells adhered poorly to DCs compared to naïve T cells in the same condition, resulting in an average ratio of *Mst1* ^*-/-*^ Treg cells and naïve T cells in each cluster ranging from 0.14 to 2.5 with a median of 0.80. To directly compare the ability of *Mst1* ^*+/+*^ and *Mst1* ^*-/-*^ Treg cells to form antigen-dependent conjugates, equal numbers of *Mst1* ^*+/+*^ or *Mst1* ^*-/-*^ Treg cells were mixed with peptide-pulsed DCs. *Mst1* ^*-/-*^ Treg cells formed conjugates with DCs at a 60% lower rate than *Mst1* ^*+/+*^ Treg cells (Figure **3B**). Thus, Mst1 is required for efficient antigen-specific contacts with DCs through LFA-1.

To examine the consequence of this defect in *Mst1* ^*-/-*^ Treg cells on contact-dependent suppressor functions, we measured the down-regulation of co-stimulatory molecules in DCs [3,4,9]. Co-culture of OT-II naïve T cells and peptide-pulsed DCs increased CD86 expression by DCs, which was almost completely abrogated by the addition of wild-type OT-II Treg cells (Figure **3C**). However, *Mst1* ^*-/-*^ Treg cells partially inhibited the CD86 up-regulation only by a half, compared to wild-type Treg cells (Figure **3C**). This result supported the notion that inefficient conjugate formation between *Mst1* ^*-/-*^ Treg cells and DCs impairs the ability of Treg cells to mediate suppressor functions.

### Defective immunological synapse formation of Mst1^-/-^ Treg cells

**Figure 4:**
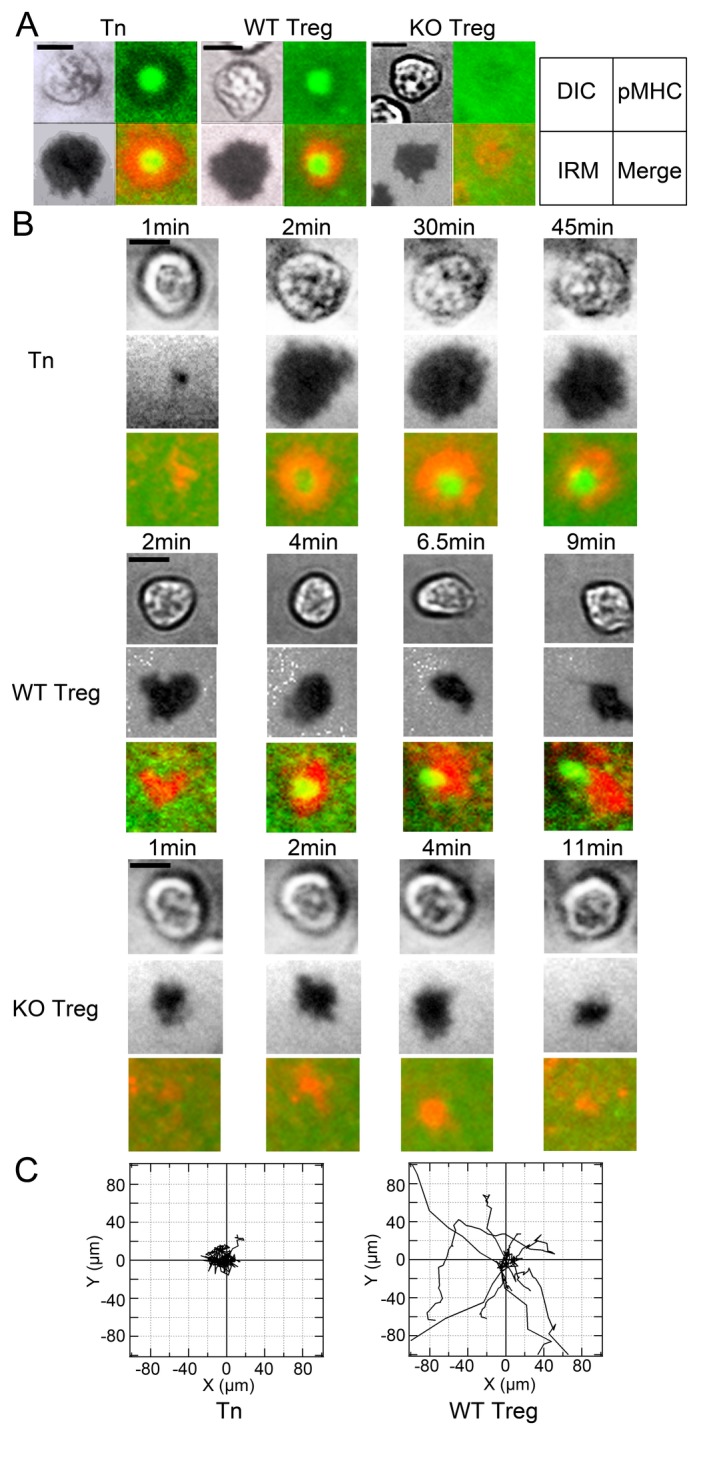
Impaired formation of immunological synapses by *Mst1* ^*-/-*^ Treg cells. (**A**) Representative images of IS in naïve T cells (Tn, left), OT-II *Mst1* ^*+/+*^Treg cells (WT Treg, middle) and OT-II *Mst1* ^*-/-*^ Treg cells (KO Treg, right) on planar lipid bilayers presenting OVA peptide/I-A^b^, CD80, and ICAM-1. DIC, IRM, and merged images of pMHC (green) and ICAM-1(red) are shown. The images represent 3 independent experiments. Bars indicate 5 µm. (**B**) Time-lapse images of IS in OT-II WT Treg and OT-II KO Treg cells on lipid bilayers. DIC, IRM, and merged views of peptide-MHC and ICAM-1 are shown. Note that the mature synapses of Treg cells were quickly transformed into mobile IS compared to that of naïve T cells. OT-II KO Treg cells failed to exhibit mature synapse. (**C**) Trajectory of Tn (left) and WT Treg (right) T cells on planar lipid bilayers. Ten representative cells were tracked and displayed on an x-y plane.

To clarify the impairment of antigen-specific contacts of *Mst1* ^*-/-*^ Treg cells with DCs, we examined IS formation using a planar lipid membrane expressing OVA peptide/I-A^b^, ICAM-1, and CD80 molecules. Foxp3^+^ OT-II Treg cells showed rapid formation of IS within a couple of minutes, characterized by a central pMHC cluster surrounding ICAM-1 and this was indistinguishable from that of naïve T cells (Figure **4A**, Video **S1**). The absence of the OVA peptide/MHC did not induce the IS formation (not depicted). Compared to naïve T cells, we found that Treg synapses were motile; symmetrical IS frequently broke by the partial loss of pSMAC with development of the leading edge and uropod (Figure **4B**, Video **S2**). Cell tracking analysis showed that naïve T cells were basically sessile, while Treg cells frequently migrated on the planar lipid bilayers (Figure **4C**). On the other hand, *Mst1* ^*-/-*^ Treg cells exhibited inefficient attachment (Figure **2S**) and poorly developed mature IS (Figure **4A, 4B**, Video **S3**). Although *Mst1* ^*-/-*^ Treg cells exhibited some ICAM-1 clusters, they did not distribute in a ring structure and pMHC clusters were absent, even with increased periods of incubation (Figure **4A, 4B**). Thus, these results clearly indicate that Mst1 is required for efficient attachment via LFA-1/ICAM-1 and development of pSMAC and cSMAC formation.

### Interactions of natural Treg cells with DCs in lymphoid tissues

**Figure 5:**
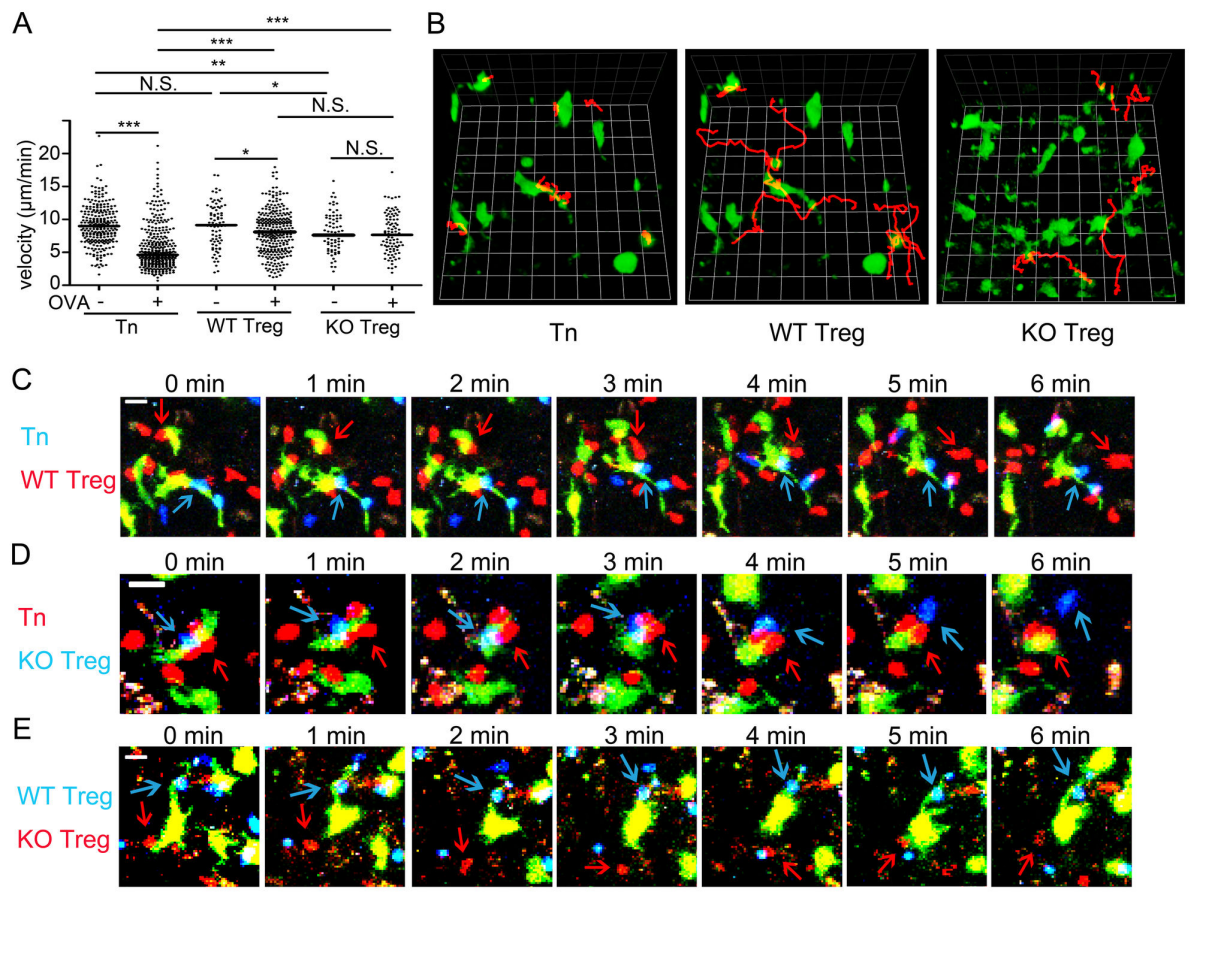
Defective interactions of *Mst1* ^*-/-*^ Treg cells with DCs within LN tissues. Migration patterns of naïve (Tn), *Mst1* ^*+/+*^Treg (WT Treg) cells and *Mst1* ^*-/-*^ Treg cells (KO Treg) in the presence of BMDCs pulsed with or without OVA_323–339_ within LN tissues were analyzed by time-lapse imaging using two-photon microscope. (**A**) Velocities of OT-II naïve T cells (Tn), WT Treg cells, KO Treg cells; Tn without OVA, n=268; Tn with OVA, n=348; WT without OVA, n=79; WT with OVA, n=290; KO without OVA, n=73; and KO with OVA, n=91. Data were pooled from 2 independent experiments. P values were calculated by Student t-test; N.S. not significant; *p<0.05, **p<0.01, ***p<0.0001. (**B**) Typical track paths of OT-II Tn cells, OT-II WT Treg cells, and OT-II KO Treg cells in the presence of pulsed DC. One column represents 15 µm. (**C**) Migration of OT-II Tn cells (blue) and OT-II WT Treg cells (red) in the presence of pulsed DCs (green). Arrows indicate stable association of Tn cells with DCs and transient-arrest of Treg cells with DC, respectively. Bars indicate 25 µm. (**D**) Migration of OT-II Tn cells (red) and OT-II KO Treg cells (blue) in the presence of pulsed DCs (green) within LN. Arrows indicate unstable contacts of KO Treg cells with DCs and bearing naïve T cells, respectively. (**E**) OT-II WT Treg cells (blue) and OT-II KO Treg cells (red) were cultured with pulsed DCs (green). WT OT-II Treg cells and KO OT-II Treg cells contacted with DCs are indicated with arrows.

Since Mst1 also regulates interstitial migration [21], the combined effects of impaired motility and IS formation may aggravate *in vivo* Treg suppression, as seen in the experimental colitis model (Figure **1**). To clarify this point, Treg cell trafficking and interactions with DCs should be examined. However, two-photon imaging of LNs with conventional intravital or explant methods after adoptively transfer of Treg cells was rather difficult due to the limited number of natural Foxp3^+^ Treg cells or induced Treg cells available from *Mst1* ^*-/-*^ mice (not depicted). To circumvent this, we took advantage of the LN slice method, which allowed us to apply relatively small numbers of Treg cells together with naïve T cells directly to the parenchyma of LNs that had been transferred with antigen-loaded DCs. Under this protocol we performed two-photon imaging to examine the interstitial migration and interaction of Treg cells with DCs within LN tissues. Foxp3^+^ OT-II Treg cells and naïve OT-II T cells were applied to the sliced tissues of peripheral LNs in which OVA-pulsed DCs had been transferred subcutaneously. Without OVA antigen, OT-II Treg and naïve T cells exhibited comparable motility with approximately 10 µm/min average velocity (Figure **5A**). In the presence of OVA antigen, naïve T cells arrested and formed stable contacts with DCs (Figure **5B, 5C**, Video **S4**). As a result, average velocity was as low as 5 µm/min (Figure **5A**). Treg cells exhibited transient arrest and swarmed around DCs bearing naïve T cells (Figure **5B, 5C**, Video **S4**); the contact duration of Treg cells was shorter than that of naïve T cells, resulting in intermediate velocities (Figure **5A**). Representative cell migration paths showed that naïve T cells were almost sessile and wild-type Treg cells exhibited a brief “stop and go” pattern (Figure **5B**). In contrast, *Mst1* ^*-/-*^ Treg cells migrated at intermediate velocities (7.6 µm/min) without the OVA antigen, indicating that Mst1 deficiency cause inefficient Treg migration (Figure **5A**). Furthermore, *Mst1* ^*-/-*^ Treg cells did not exhibit arrest even in the presence of antigen; *Mst1* ^*-/-*^ Treg cells made contacts with DCs, but were easily dissociated and apart from them (Figure **5B, 5D** **, 5E,** Video **S5** **-6**). As a result, there was no significant difference in velocity with or without antigen (Figure **5A**). Thus, the Mst1 deficiency had detrimental effects on both motility and antigen-specific arrest within lymphoid tissues. These combined defects likely explain the severe impairments of *Mst1* ^*-/-*^ Treg cells in the suppression of experimental colitis.

## Discussion

Here we showed that *Mst1* ^*-/-*^ Treg cells failed to prevent the development of experimental colitis and antigen-specific suppression of naïve T cells proliferation *in vitro*. *Mst1* ^*-/-*^ Treg cells exhibited defective interactions with DCs, resulting in impaired down-regulation of costimulatory molecules. While wild-type CD4^+^ Foxp3^+^ Treg cells formed mobile immunological synapses on supported planar membrane, *Mst1* ^*-/-*^ Treg cells exhibited reduced attachment and did not elicit pSMAC and cSMAC formations. Consistently, antigen-specific wild-type Treg cells exhibited dynamic mobile contacts with antigen-pulsed DCs bearing stably associated naïve T cells. In contrast, *Mst1* ^*-/-*^ Treg cells were defective in their interactions with DCs. Thus, Mst1 is required for Treg cells to mediate contact-dependent suppressor functions. Since Mst1 is required for medullary migration and self-antigen recognition of mature thymocytes by regulating LFA-1/ICAM-1 interaction [24], LFA-1 regulation seems to be a basic regulatory mechanism by Mst1 that controls adhesion and migration of Treg cells.

It should be noted that natural Treg cells and naïve T cells within LN tissues exhibit a distinct interaction pattern of short-lived contacts and stable contacts with DCs, respectively. This difference is corroborated by mobile IS of Treg cells on a supported planar membrane. The short-lived mobile contacts did not show preferential clustering of Treg cells with DCs in the LN tissues, which was in contrast to the *in vitro* situations that lacked motility-support environments. Taken together, these observations indicate that short-lived mobile behavior is characteristic of natural Treg cells within LN environments. Considering this aspect of Treg cell interactions with DCs, it is unlikely that Treg cells inhibit naïve T-cell priming by occupying DCs in LNs, but rather via down-modulation of co-stimulatory properties or other short contact-dependent suppression such as perforin-dependent killing of DCs mechanisms [5].

Our results show a novel role of *Mst1* in the process of IS formation by natural Treg cells. We previously showed that Mst1 and RAPL were localized in vesicle compartments and relocated to the T-APC contact sites with co-localization with LFA-1 [20,32]. The current study extends the previous studies and indicate that a linkage of spatial organization of LFA-1/ICAM-1 and TCR/pMHC bindings. Talin1 deficient T cells exhibits unstable T-dC interactions with impaired polarization of vinculin and F-actin at the IS. However, LFA-1 is still recruited to the contact sites with DC [19]. Therefore, talin stabilizes the IS formation through a linkage of LFA-1 to F-actin, whereas recruitment of LFA-1at the contact site is a talin-independent process that requires Mst1.

The mechanism by which Mst1 controls cSMAC formation is not clear. Since the IS formation can be correlated with topologically driven receptor segregation based on the size of the different receptor -ligand pairs [33], impaired large LFA-1/ICAM-1 cluster formation could also impair small TCR/peptide-MHC segregation. TCR microclusters formed in the periphery of SMAC are centripetally transported via the processes requiring dynein/microtubule [34] and myosin IIA/F-actin [35], or transported toward actin-depolymerization sites [36]. Mst1 deficiency could affect these processes. The recent study reported that Dock8 was one of the Mst1 target upon chemokine stimulation, and double deficiency of Mst1 and Mst2 impaired chemokine-stimulated F-actin development with reduced Rac and Rho activities [23]. *Dock8* ^*-/-*^ CD8 T were reported to be defective in ICAM-1 accumulation at the contact site with antigen-loaded DCs [37], which suggest that Dock8 is involved in IS formation. Dock8 is a Cdc42 guanine nucleotide exchange factor, which regulates actin polymerization at the leading edge of DCs and plays a crucial role in interstitial migration of DCs [38]. Thus, Mst1 signals could control cytoskeletal signals involved in cSMAC formation via Dock8 activation. Further studies are needed to clarify the role of Dock8 in Mst1-regulation of LFA-1 in Treg cells.

Treg cells dynamically interact with antigen-loaded DC *in vitro* [9]. Our results demonstrate dynamic adhesive behavior of antigen-specific Foxp3^+^ natural Treg cells with antigen-loaded DC within LN tissues, which is distinct from stable adhesion of naïve T cells with DC. The stable OT-II naïve T cell IS within 30 min observation time is in agreement with those of naïve AND or OT-I T cells, which showed stable IS or IS with limited motilities (1.5 µm/min) [16], followed by exhibiting mobile IS at later time points (90min or later) [36]. The OT-II Treg IS formation on the supported planar membrane was indistinguishable from naïve OT-II T cells at the early step, however was quickly transformed into the mobile IS. Notably, Zanin-Zhorov A et al. has recently reported that human CD4^+^ CD25^+^ Treg IS exhibited relatively more stable on the supported planar bilayers presenting anti-CD3 mAb and ICAM-1, compared to effector T cells [17]. Although the comparison between human CD4^+^ CD25^+^ Treg cells and mouse Foxp3^+^ Treg cells under the same condition is not available, the disparity to their study might reflect differences in TCR stimulation, Treg subsets, or distinct regulation of mouse and human T cells.

Previous studies showed that the presence of Treg cells inhibited stable contact between antigen-specific naïve T cells and DCs within LN tissues [5–8]. However, in our experimental conditions of 20–30 min observation time, naïve T cells arrested normally and formed conjugates with DCs in the presence of Treg cells. This could be due to the longer period of time required by Treg cells to down-modulate co-stimulatory molecules on DCs and/or the ability of the high-affinity OT-II TCR override the inhibitory effect of Treg cells on the stable contacts. Indeed, low-affinity, but not high-affinity interactions of OT-I T cells with DCs was affected by antigen-specific Treg cells [8].

CD4^+^ Foxp3^+^ Treg cells were reduced in the thymus but recovered in the periphery in *Mst1* ^*-/-*^ mice [24]. We noticed that the *in vitro* induction of Foxp3^+^ Treg cells from CD4^+^ naïve T cells with TGFβ was less efficient compared to wild type. However, the levels of Foxp3 and CTLA-4 of induced *Mst1* ^*-/-*^ Treg were comparable with intact suppressor functions on T cell proliferation induced by TCR stimulation. The inefficient induction of Treg cells from *Mst1* ^*-/-*^ CD4^+^ naïve T cells suggest that Mst1-regulation of homotypic cellular interactions through LFA-1/ICAMs is involved in the Treg induction processes *in vitro*, which could be compensated *in vivo*.

Mst1-deficient mice developed lymphocyte and plasma cell infiltrations into multiple organs with hypergammaglobulinemia and autoantibody productions with age. Combined defects of Treg cells and thymic selections [24] likely underlie impaired self-tolerance in Mst1-deficient mice. Our study sheds a new insight into autoimmunity-like phenotypes with T cell immunodeficiency in Mst1-deficient human [39,40], and could aid in the development of Treg manipulation and therapeutics.

## Supporting Information

Figure S1 **Expression of surface molecules on Mst1^-/-^ Treg cells.** Expression of Foxp3, CD25, GITR, CTLA4, CD11a, CD18, and CCR7 in Mst1^+/+^ Treg cells and Mst1^-/-^ Treg cells. The histograms represent 3 independent experiments with 3 mice per genotype.
(TIF)

Figure S2 **Defective adhesion of *Mst1*^*-/-*^Treg to ICAM-1.** OT-II FoxP3 ^+^ CD4^+^ Treg cells prepared from *OT-II*; *Foxp3-GFP*;*Mst1* ^*+/+*^ (WT) or *OT-II*; *Foxp3-GFP*;*Mst1* ^*-/-*^ (Mst1-KO) mice were incubated on supported planner lipid bilayer presenting peptide-MHC, ICAM-1 and CD80 as described in Materials and Methods. The cell attachment was measured using interference reflection (IR) images. The levels of attached cells was calculated by dividing the number of IR-positive cells with the total number of cells in each field. The data are collected from four independent fields per each cell type.
(TIF)

Video S1 **Time-lapse imaging of IS formation using naïve T cells.** Formation of IS by naive T cells on a planer lipid membrane loaded with OVA peptide MHC, ICAM-1 and CD80. DIC (upper left), Alexa488-labeled OVA peptide/I-Ab (upper right), IRM (lower left), a merged image of Alexa488-labeled OVA peptide/I-Ab and ICAM-1-Cy3 (lower right). Bars indicate 5µm.
(MOV)

Video S2 **Time-lapse imaging of IS formation using *Mst1*^*+/+*^ Treg cells.** Formation of IS by *Mst1* ^*+/+*^ Treg cells on a planer lipid membrane loaded with OVA peptide MHC, ICAM-1 and CD80. DIC (upper left), Alexa488-labeled OVA peptide/I-Ab(upper right), IRM (lower left), a merged image of Alexa488-labeled OVA peptide/I-Ab and ICAM-1-Cy3 (lower right). Bars indicate 5µm.
(MOV)

Video S3 **Time-lapse imaging of IS formation using *Mst1*^*-/-*^ Treg cells.** Formation of IS by *Mst1* ^*-/-*^ Treg cells on a planer lipid membrane loaded with OVA peptide MHC, ICAM-1 and CD80. DIC (upper left), Alexa488-labeled OVA peptide/I-Ab (upper right), IRM (lower left), a merged image of Alexa488-labeled OVA peptide/I-Ab and ICAM-1-Cy3 (lower right). Bars indicate 5µm.
(MOV)

Video S4 **Two-photon imaging of naïve T cells and *Mst1*^*+/+*^ Treg cells in LN tissues.** Time-lapse video of OT-II naïve T cells (blue) and *Mst1* ^*+/+*^ OT-II Treg cells (red) interacting with OVA323–339-pulsed DCs (green) in LN tissues. LPS-stimulated, OVA peptide-pulsed BMDCs subcutaneously injected into C57BL/6 mice. After 24 h, brachial LNs were isolated and cut open, followed by direct application of the mixture of CMAC- labeled OT-II naïve T cells and CMTMR-labeled *Mst1* ^*+/+*^ or *Mst1* ^*-/-*^ OT-II Treg cells to the cut LN sliced tissues and time-lapse images were taken by two-photon microscopy as described in Methods. Bars indicate 25µm.
(MOV)

Video S5 **Two-photon imaging of naïve T cells and *Mst1*^*-/-*^ Treg cells in LN tissues.** Time-lapse video of OT-II naïve T cells (red) and *Mst1* ^*-/-*^ OT-II Treg cells (blue) interacting with OVA323–339-pulsed DCs (green) in LN tissues taken by two-photon microscopy as in Video S4. Bars indicate 25µm.
(MOV)

Video S6 **Two-photon imaging of *Mst1*^*+/+*^ and *Mst1*^*-/-*^ Treg cells in LN tissues.** Time-lapse video of *Mst1* ^*+/+*^ OT-II Treg (blue) and *Mst1* ^*-/-*^ OT-II Treg cells (red) interacting with OVA323–339-pulsed DCs (green) in LN tissues taken by two-photonmicroscopy as in Video S4. Bars indicate 25µm.
(MOV)
